# Effectiveness of dupilumab for chronic prurigo in elderly patients with atopic dermatitis^☆^

**DOI:** 10.1016/j.abd.2022.01.011

**Published:** 2022-11-11

**Authors:** Shinji Mitsuyama, Tetsuya Higuchi

**Affiliations:** Department of Dermatology, Sakura Medical Center, School of Medicine, Toho University, Chiba, Japan

Dear Editor,

Chronic prurigo (CPG), such as prurigo nodularis, is often a complication of atopic dermatitis (AD).^1^ CPG is a common and distinct skin disease characterized by multiple pruriginous skin lesions,^2^ and its pathophysiological mechanisms remain unknown; however, the involvement of an atopic predisposition has been suggested.^2^ CPG in AD is highly intractable to traditional treatments. Herein, we present the cases of four elderly patients with AD complicated by CPG in whom traditional treatments had failed previously and describe their successful treatment with dupilumab within the last 2 years.

The clinical characteristics of the four patients are shown in Table 1. The AD in the four patients was complicated by CPG (Fig. 1A). None of the patients had a history of childhood AD, but the onset of AD was noted in old age. As previous traditional treatments had failed in all the patients, dupilumab treatment was initiated at standard doses of 600 mg subcutaneously at week 0 and then at 300 mg every other week. All the patients showed significant improvement in pruritus 2‒4 weeks after initiation of dupilumab treatment. In all cases, treatment with dupilumab was very effective, and the Eczema Area and Severity Index (EASI)-90 was achieved 4‒8 weeks after initiating dupilumab treatment (Fig. 1B). Although all the patients had various medical diseases, no side effects were observed in any patient.

**Table 1:** Clinical characteristics of four elderly patients with atopic dermatitis complicated by chronic prurigo.

Clinical characteristic	Case 1	Case 2	Case 3	Case 4
Age of first visit	71-year-old	65-year-old	84-year-old	69-year-old
Sex	Male	Male	Male	Female
Age of onset of AD	67	65	84	67
Age of patient when dupilimab was initiated	73	66	85	70
History of childhood atopic dermatitis	No	No	No	No
Complication	Allergic rhinitis, Allergic conjunctivitis, Type 2 diabetes, Diabetic nephropathy, previous Myocardial Infarction	Allergic rhinitis, Bronchial asthma, Cerebral infarction	Hypertension, Dyslipidemia, Sleep apnea syndrome, Bilateral osteoarthritis of the hip	Bronchial asthma, Allergic bronchopulmonary aspergillosis, Eosinophilic pneumonia, Hypertension, Type 1 diabetes mellitus, Hashimoto's thyroiditis, Ulcerative colitis
Previously failed therapies	H1 antihistamines, Topical corticosteroids, NB-UVB phototherapy, Prednisolone	H1 antihistamines, Topical corticosteroids, NB-UVB phototherapy, Prednisolone, Cyclosporine A	H1 antihistamines, Topical corticosteroids, NB-UVB phototherapy, Prednisolone	H1 antihistamines, Topical corticosteroids, NB-UVB phototherapy, Prednisolone
Histological findings	Spongiosis, Perivascular infiltration of lymphocytes and eosinophils in the upper dermis	Spongiosis, Perivascular infiltration of lymphocytes and eosinophils in the upper dermis	Epidermal hyperplasia, Spongiosis, Perivascular infiltration of lymphocytes and eosinophils in the upper dermis	Hyperkeratosis, Epidermal hyperplasia, Spongiosis, Perivascular infiltration of lymphocytes and eosinophils in the upper dermis
Percent BSA affedted when dupilimab was initiated (%)	17	52	56	57
EASI score when dupilimab was initiated	16.3	29	20.7	17.3
Total IgE (IU/mL)	3,100	4,300	190	7,900
TARC (pg/mL)	1,050	5,500	12,800	4,050
Absolute eosinophil count (/μL)	678	1,410	1,782	1,335a

AD, Atopic Dermatitis; BSA, Body Surface Area; EASI, Eczema Area and Severity Index; H1, Histamine 1; NB-UVB, Narrow-Band Ultraviolet B; TARC, Thymus and Activation‐Regulated Chemokine.

**Figure 1:**
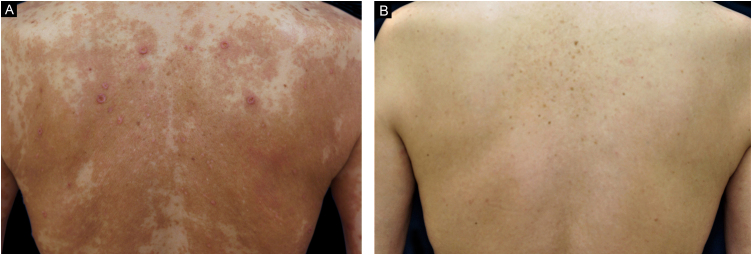
Clinical presentation of the upper back in a 65-year-old Japanese man (Case 2). (A) Pruritic lichenified plaques, papules, and prurigo nodules were present before dupilumab treatment. (B) Complete clearance of the cutaneous manifestations was achieved by 24 months after the initiation of dupilumab.

Dupilumab is a fully human monoclonal antibody directed against the α subunit of the Interleukin (IL)-4 receptor; it inhibits the signaling of IL-4 and IL-13 pathways, which play pivotal roles in the pathogenesis of Th2 inflammation and AD. Dupilumab is reportedly effective for CPG in patients with AD.^3^, ^4^

Our patients with CPG in AD showed highly elevated total IgE and thymus and activation-regulated chemokine levels and peripheral blood eosinophil counts. Histological findings revealed superficial, perivascular, and interstitial eosinophil infiltration in the skin lesions in all the patients, which corresponds with the characteristic histological findings reported in CPG ‒ a superficial perivascular and interstitial inflammatory infiltration composed mainly of lymphocytes and eosinophils identified in the skin lesions.^5^ According to the literature, dupilumab is also effective for eosinophilic diseases, such as eosinophilic pneumonia,^6^ eosinophilic chronic rhinosinusitis,^7^ and eosinophilic esophagitis.^8^ Eosinophilic pneumonia in the 69-year-old female patient was improved by dupilumab treatment. We presume that our cases pathologically involved both Th2 response and eosinophilic inflammation and that dupilumab was effective in managing both these conditions.

In elderly patients with AD, systemic therapy with immunosuppressive agents is difficult due to various complications. Additionally, topical steroid treatment is difficult due to skin atrophy caused by aging. Generally, dupilumab is well tolerated, with few adverse effects. Therefore, dupilumab is a useful treatment option for CPG in elderly patients with AD.^3^ Because our patients were elderly, it was difficult to continue systemic therapy and topical steroids due to various complications and skin atrophy. Therefore, we initiated dupilumab treatment with successful in treating the patients.

Our study suggests the usefulness of dupilumab for CPG in elderly patients with AD.

## Financial support

None declared.

## Authors' contributions

Shinji Mitsuyama: Approval of the final version of the manuscript; Study conception and planning; Critical literature review; Data collection, analysis, and interpretation; Preparation and writing of the manuscript.

Tetsuya Higuchi: Approval of the final version of the manuscript; Critical literature review; Manuscript critical review.

## Conflicts of interest

None declared.
